# Poly(Glycerol Sebacate)–Poly(l-Lactide) Nonwovens. Towards Attractive Electrospun Material for Tissue Engineering

**DOI:** 10.3390/polym11122113

**Published:** 2019-12-16

**Authors:** Piotr Denis, Michał Wrzecionek, Agnieszka Gadomska-Gajadhur, Paweł Sajkiewicz

**Affiliations:** 1 Laboratory of Polymers and Biomaterials, Institute of Fundamental Technological Research, Polish Academy of Sciences, Pawińskiego 5B Street, 02-106 Warsaw, Poland; psajk@ippt.pan.pl; 2 Faculty of Chemistry of Warsaw University of Technology, Noakowskiego 3 Street, 00-664 Warsaw, Poland; mwrzecionek@ch.pw.edu.pl

**Keywords:** electrospinning, degradable polymers, synthesis, structure, crosslinking

## Abstract

Two types of poly(glycerol sebacate) (PGS) prepolymers were synthesized and electrospun with poly(l-lactic acid) (PLA), resulting in bicomponent nonwovens. The obtained materials were pre-heated in a vacuum, at different times, to crosslink PGS and investigate morphological and structural dependencies in that polymeric, electrospun system. As both PGS and PLA are sensitive to pre-heating (crosslinking) conditions, research concerns both components. More interest is focused on the properties of PGS, considering further research for mechanical properties and subsequent experiments with PGS synthesis. Electrospinning of PGS blended with PLA does not bring difficulties, but obtaining elastomeric properties of nonwovens is problematic. Even though PGS has many potential advantages over other polyesters when soft tissue engineering is considered, its full utilization via the electrospinning process is much harder in practice. Further investigations are ongoing, especially with the promising PGS prepolymer with a higher esterification degree and its variations.

## 1. Introduction

It has already been nearly two decades since poly(glycerol sebacate) (PGS) started to draw attention in terms of fundamental research and its usefulness in a variety of biomedical applications. PGS is classified as a biodegradable elastomer, potentially useful in soft tissue engineering and regenerative medicine applications. The first significant publication devoted to the use of PGS in tissue engineering was published in 2002 in Nature Biotechnology [1]. It was a time when tissue engineering was already being actively developed with an extensive search for new biomaterials for cell cultures aiming at the design of new implants and stents for improving human life. This publication was actually a report on how to obtain that new polymeric material, as well as its main advantages.

A few general words of introduction shall be written about biodegradable polymers, which are important in different fields of bioengineering. The properties of biodegradable polymers investigated in model conditions, including biological functionality in vitro conditions, can be far from those observed for materials placed in a dynamically changing in vivo environment, subjected to various, complex interactions, including mechanical and enzymatic interactions. In addition, biomaterials interacting with the surrounding tissues should irritate tissues as little as possible, not provoking local inflammation. Ideally, the biomaterial, or rather the constructs created from it, can be perceived as such with properties close to the natural extracellular matrix (ECM), surrounding cells, and tissues. The ECM can be described as a soft, but robust and flexible fibrous protein network immersed in a viscoelastic gel that is rich in proteoglycans, providing mechanical stability and structural integrity for tissues and organs. In this context, a soft, biodegradable elastomer that easily returns to its original form after a relatively large deformation would be beneficial. 

In the aforementioned pioneer publication by Y. Wang et al. [1], three major classes of biodegradable elastomers were mentioned—hydrogels, peptides similar to elastin, and polyhydroxyalkanoates (PHA). PGS was then presented as an inexpensive, biodegradable, and biocompatible elastomer. Compared with hydrogels, its durability was much higher. Compared with elastic-like peptides obtained by bacterial fermentation, it was expected to be inexpensive, non-immunogenic, and free from endotoxins. In addition, PGS was supposed to have the capacity for much larger reversible deformations than the “supposedly” elastomeric variant of PHA/poly-4-hydroxybutyrate (P4HB).

There is some analogy between PGS and vulcanized rubber. Both materials form a crosslinked, three-dimensional amorphous structure consisting of entangled macromolecules. In nature, similar solutions can be found on the way to create durable materials with elastomeric properties. Elastin and collagen—the main protein and fibrous components of the extracellular matrix—are also present in nature in a form of crosslinked material. In addition to covalent crosslinking, the hydrogen bonds formed by hydroxyproline hydroxyl groups are also responsible for stabilizing of the collagen fibrilar structure, and hence the mechanical strength. In collagen fibril, there are two types of hydrogen-bonded interactions. The first is a direct interprotein hydrogen bond between a hydroxyproline hydroxyl group in one tropocollagen and a glycine carbonyl group in its neighbour. The second is a bridging water molecule, which links two adjacent tropocollagens by forming hydrogen bonds to each of them simultaneously, linking the two proteins via the hydroxyproline hydroxyl group in each one. Hence, for example, weakening of collagen fibers when the production of hydroxyproline in the body is impaired. Correctly functioning collagen fibers are capable of up to 20% reversible deformations, which is much more than for common biodegradable polymers such as polycaprolactone (PCL), polylactide (PLA), polyglycolide (PGA), or PLGA copolymer. Reversible deformation of collagen is also greater than in the mentioned P4HB, for which the given value is usually about 10%.

For the research group who first synthesized PGS polymer, there were two fundamental hypotheses and several criteria [1]. The first hypothesis assumed that good mechanical properties could be attained by covalent crosslinking and hydrogen bonding. The second hypothesis was to obtain rubber-like elastic properties by producing a three-dimensional network of entangled macromolecules via copolymerization, where at least one of the monomers is trifunctional.

The following criteria were intended to characterize obtained material [1]:

- Hydrolysis as a degradation mechanism and hydrolysable ester bonds;
- Adequate degree of crosslinking, providing elastomeric properties;
- Chemical bonds that are involved in crosslinking shall be hydrolysable and be identical to those present in the main chain to minimize heterogeneous degradation;
- The monomers from which the material is synthesized should be non-toxic, at least one of them should be trifunctional, and at least one of them should provide hydroxyl groups for hydrogen bonds.

Investigations of PGS and its application-potential are often limited by inconsistency in reproducing process conditions, which was pointed by a group of researchers from Canada [2]. The inconsistency may come from the volatile nature of glycerol during the esterification at a low pressure and high temperature, which are the typical conditions for PGS synthesis. Inconsistence in the synthesis process may even come from subtle variations in equipment. Authors [2] suggested that the degree of esterification could be used to precisely predict the properties of PGS materials. It was pointed out also that the quick method of PGS synthesis with microwave-assisted prepolymerization does not show noticeable differences in terms of chemical bonds compared with a conventional prepolymerization method in a nitrogen oven. The microwave method provides a high degree of esterification in a much shorter time. In terms of tailoring PGS mechanical properties, authors suggested the addition of crosslinking agents such as methylene diphenyl diisocyanate (MDI), which provides a higher Young’s modulus, preserving deformability at the same time [2]. 

Clear presentation of the correlation between parameters of synthesis (including glycerol and sebacic acid molar ratio), subsequent curing, and physicochemical properties of PGS were shown, for example, in the works of [2,3]. “Gold standard” proposed in the literature [2,3] is the equimolar reagents ratio and curing at around 130 °C–135 °C for 48 h. In terms of molar ratios, a higher polyol content results in viscous, sticky, and generally difficult to handle materials. Predominance of acid may lead to rapid mass loss by the hydrolysis mechanism, but can be considered in specific applications. A time of 24 h of curing is usually considered as too short to obtain dense, non-swellable, well-crosslinked (with more than 85% chains crosslinked) material. 

### PGS—Good Properties for Soft Tissues

Soft tissues, especially in the cardiovascular system, were a natural point of interest in the elastomeric properties of surface-degradable PGS or materials modified with PGS. Lower stiffness and higher reversible deformations, which can be kept for longer time compared with materials that exhibit bulk degradation, were seen as a very attractive perspective.

In the field of soft tissue engineering, poly(dl-lactide-co-glycolide) (PLG) is often mentioned as a well-known, nearly reference material; thus, performance of PGS in a physiological environment was sometimes compared to properties of PLG or other copolymers of aliphatic polyesters. In 2002, a Korean group investigated PGS in terms of in vivo degradation characteristics, comparing it to PLG [4]. The most important observation was that PGS degrades primarily by surface erosion, while PLG degrades primarily by bulk degradation. Degradation profiles of changes in weight and in mechanical strength were linear for PGS. Similar characteristics were observed for preservation of the geometry of material during the degradation time. During in vivo degradation tests, PGS lost at least 70% of mass after 35 days of experiment. Water content of PGS implants rose linearly during the experiment up to 15%, which is much lower compared with PLG. 

Another study of PGS properties performed also in vivo conditions was published by Sundback et al. in 2005 [5]. The hypothesis that motivated investigators was that PGS may be attractive as a nerve guide material. Tissue response profile and in vitro tests using Schwann cells (adherence, proliferation, apoptosis), together with biocompatibility results, were all promising. In addition, inflammatory response to PGS decreased with time owing to gradual resorption compared with the high inflammatory response for PLGA, which was related, in the authors’ opinion, to the rapid mass loss of PLGA.

The group of scientists from London published results in 2008 devoted to developing of biocompatible, degradable heart patch with high elasticity [6]. Potential biomaterials for heart tissues engineering including PGS are presented in Table 1.

Chen et al. [6] chose three temperatures in which PGS was synthesized by polycondensation—110, 120, and 130 °C—reporting large changes of Young’s modulus, being 0.056, 0.22, and 1.2 MPa, respectively. According to the authors, that range should satisfy the mechanical requirements of heart patches or other myocardial constructs. Authors claimed that PGS synthesized at 110 °C had a similar Young modulus to heart muscle at a lower strain region, while the Young modulus of PGS synthesized at 120 °C was similar to the heart muscle at a higher strain region.

Even though formation of PGS fibers by electrospinning may not be the favorite technique among researchers, S. Ramakrishna et al. published in 2013 the paper devoted to biomaterials for myocardial infarction formed by the core-shell electrospinning method [7]. PGS (core)/collagen (shell) fibers were successfully electrospun, with mechanical and biological properties tailored towards soft tissues of the heart. The main structural protein of myocardium is collagen type I, providing proper stiffness and support, while type III provides flexibility. PGS, used as a core, brought more elastomeric behavior compared with collagen-only scaffolds and provided, together with the collagen-shell, a desirable elastic modulus (circa 4.2 MPa), comparable to native myocardium. Cells’ proliferation was not much, but noticeably higher for collagen–PGS scaffolds compared with collagen scaffolds. Another group of researchers pointed out that, in soft tissue engineering, PGS may not fulfill its function because of limited water uptake capacity (~2%) [8]. They suggested structural/chemical modifications by synthesis of amphiphilic block PGS–polyethylene glycol (PGS-PEG) copolymers to obtain more favorable water uptake and retention characteristics. As a result, authors not only achieved a significant increase in water uptake by PGS, but also noticed a slight improvement in its mechanical stability under dynamic loading. Both for pure PGS and with a higher PEG content (40% and 60%), during tensile tests, the material displayed high recoverability (usually >95%) and negligible deformation hysteresis, with the exception of sample containing 20% PEG. It is worth mentioning that cyclic tests were performed only for five cycles. Typically, for elastomers, all samples exhibited a plateau in energy absorbed and recovery of the network after the first cycle, but it would be really useful to know the behavior of these materials after multiple cycles, dozens or hundreds at least, especially considering the application potential. With preserved elastomeric properties, increased elongation abilities, and tunable Young modulus in a wide range (13 kPa–2.2 MPa, depending on PEG contribution; more PEG = lower modulus), authors created a new kind of potential biomaterial for soft tissue engineering. The in vitro degradation rate also increased with PEG contribution and seemed to be the most important factor to be considered. Pure PGS is marked by ca. 10% mass loss after 21-day-long in vitro degradation. Addition of 20% of PEG increases that value to 20% level (double increase), while with 60% addition of PEG, the mass loss leaps toward 80% after 21 days. It should be critical to consider such properties of suggested copolymers, as in vivo conditions would most probably bring even much faster degradation rates, considering susceptibility of PGS to enzymatic degradation. Considering many publications, where authors, incorporating PGS, aimed to provide material for soft tissues engineering, it can be already confirmed that PGS is a promising material in that field. From the fundamental point of view, there is still noticeable shortage of mechanical test results, especially considering PGS’s elastomeric nature, and its capability for reversible deformations. The limit of such deformations is not clear, nor are the limitations, when such properties are possible or impossible to achieve.

The first and only comprehensive summary on PGS was published in 2012 by Rai et al. [9]. The authors, in a detailed manner and with diligence, covered a wide range of information about PGS. Starting from synthesis, through properties (physical, chemical, mechanical, thermal), structure (crystallinity) up to degradation behavior, and its biocompatibility. A significant part of the review was dedicated to the use of PGS in medical applications, especially within the scope of soft tissue engineering. Selected processing technologies and modifications of PGS (composites, blending, functionalization) were also presented in the paper. Concluding remarks showed PGS in a positive light, with emphasis on its increasing importance in the biomedical field and features like tailorable mechanical properties (within soft tissues properties range) and degradation kinetics. Hard tissue engineering seemed to be not obvious, but an area with relative potential for new applications for PGS. The need for utilization of various processing methods including electrospinning was suggested on the way to obtain PGS scaffolds with structure targeted towards specific necessities. That was probably the main reason to embed the fundamental research of our investigations in the electrospinning area, considering it, with the support of our experience, as promising. PGS in prepolymer form is an oligomer with molecular weight usually in a range of 5 kDa–12 kDa, which does not provide sufficient viscosity to be electrospun singly. Even slightly crosslinked PGS is considered as an inappropriate polymer for the electrospinning process, as its highly viscous form does not compose nonwoven, but rather film.

The following is known to authors about PGS electrospinning:

- PGS cannot be electrospun singly, neither in pre-polymer form, nor in crosslinked form. It was highly probable that overcoming this limitation is possible by electrospinning of only slightly crosslinked prepolymer with a higher molecular weight, but still soluble. However, it is already known by authors that the slightly crosslinked PGS form is highly viscous and, without any additives, non-electrospinnable.
- One of the feasible methods is to process it in core-shell geometry, with PGS prepolymer as a core. During the subsequent process—crosslinking followed by leaching—the shell polymer must be thermally stable in the crosslinking temperature range (120 °C–140 °C).
- The other method is to electrospin a blend of PGS pre-polymer with the so-called carrier polymer, (e.g., PVA), followed by curing and leaching out the carrier polymer and residuals of non-crosslinked pre-PGS; such electrospinning of blends may be more reliable and much easier to optimize than core-shell. The drawbacks are related to leaching of the carrier polymer in itself, the residuals of leached polymer, and unknown influence of interactions between PGS and the carrier polymer on PGS crosslinking.
- Electrospinning of pre-PGS as a bicomponent blend with, for example, PCL or PLA or other components, without subsequent leaching, is another promising option. It is a blend-electrospinning, with an intention that PGS prepolymer will bring novel properties, that is, hydrophilicity, with a possible effect on mechanical properties, particularly when PGS contribution is the majority. In the case of blends with PCL, PGS cannot be crosslinked, so there is an essential lack of possibility for elastomeric properties, leading to a question of whether the whole idea is worth the effort. There are other, simpler methods of increasing hydrophilicity rather than synthesis of PGS and electrospinning with addition of PCL. PLA may be a more appropriate choice—its melting point is around 160 °C–180 °C, so the PGS in such blends can be crosslinked. Such investigations are being undertaken by the authors of this publication, using various contributions of PGS in blends with PLA, ranging between 25% and 75%.

Even though, in a review from 2012 [9], electrospinning was not favored among other processing methods, various approaches of using electrospinning are known in the recent history of PGS. That review article might be even considered as a turning point in using the electrospinning method in PGS processing. Previous attempts were rather preliminary, and they were presented in such way in the review—the electrospinning method was not suggested then, but of course, was seen as full of potential, like almost everything at preliminary stage. It was briefly summarized in the introduction to one of the publications about electrospinning of the PGS–PCL blends [10].

Probably, one of the first attempts of PGS electrospinning was published in 2008 [11]. The core-shell electrospinning method was used, with PGS as a core, and poly(l-lactide) (PLLA) as a shell. Authors tried several times to electrospin fibers directly from polymerized PGS or PGS prepolymer, without positive results. Thus, PLLA was the electrospun (shell) material, and the blend of PGS and PLLA was constrained inside. The blend was used to obtain desired viscosity for the process. After electrospinning, PGS was cured and the PLLA shell was removed before cellular tests. Synthesis of PGS was held with the 1:1 ratio of glycerol and sebacic acid. Electrospun fibers were cured in 130 °C in vacuum, for 24 h. Dichloromethane was used to remove the PLLA shell, and Fourier-transform infrared spectroscopy (FTIR) analysis confirmed that most of PLLA was washed out. Cellular compatibility tests were done using human dermal microvascular endothelial cells (HDMEC). After 48 h of in vitro incubation, authors achieved around 95% cellular viability with good cells attachment to the scaffold.

In the year 2010, one of the first publications about PGS–PCL blends electrospinning appeared [12]. Such a composition and approach is intriguing though. To electrospin such a blend in a common solvent (chloroform–ethanol mixture), a PGS prepolymer with relatively low molecular weight (in that case, 12,000 Da) needs to be used. The relatively low temperature of PCL melting also restricts further processing of such materials, and thus PGS cannot be crosslinked thermally. Such modification with non-crosslinked PGS brings a positive impact on cellular tests, with increased wettability compared with pure PCL. However, in such a case, the elastomeric character, being important for several biomedical applications, is not reached.

The following years brought many publications in which electrospun PCL–PGS blends were investigated within fundamental and utilitarian scopes, starting from studies of structure and mechanical properties of nonwovens and fibers, through cell guidance and corneal tissues, until materials for heart valves and cardiac patches [10,13,14,15,16,17]. Such approaches, where PGS prepolymer without subsequent crosslinking is reinforced by much tougher polyester with very good mechanical properties, and at the same time is carried by that polymer during the electrospinning process, may provoke thoughts that the elastomeric potential of PGS is wasted. On the other hand, the PGS prepolymer compared with crosslinked PGS is hydrophilic and degrades faster via hydrolysis. At the same time, it may promote cells’ attachment to scaffolds and their growth. Fibrous topography does not affect cells’ viability, and by the morphological orientation of fibers, the alignment of cardiomyocytes can be successfully promoted. So, the general conclusion is that, from the tissue engineering perspective, even PGS in prepolymer form may act as a desirable and functional material.

In 2015, a group from Pittsburgh published a very interesting paper about electrospinning of PGS [18], with subsequent crosslinking. PVA, which is non-toxic, was used as a carrier polymer, blended with PGS prepolymer. Electrospun nonwoven was thermally crosslinked at various crosslinking conditions, and PVA was removed by leaching in water, while PGS prepolymer residuals were removed by leaching in ethanol. As a result, elastomeric scaffold was obtained, as authors claimed. There were some limitations in the proposed method, like PVA residuals in the final product and relatively small pores within the nonwovens. Another limitation in the interpretation of those results was a lack of mechanical tests that would show that there is indeed an elastic, reversible deformations range, when the material is subjected to stretching. Cyclic tests would be preferred to be presented.

The recent three years brought a few more publications from the scope of PGS electrospinning, with a focus on applications for soft tissue engineering and nerve tissue engineering [19,20,21,22,23,24,25], and a few more about the electrospinning process with PGS as one of the components [26,27,28,29,30,31]. None of them, however, were devoted to investigations of electrospinning of PLA–PGS blends. In this article, we focus on electrospinning of PLA–PGS blends. This approach is not popular, not explored much, generates many problems, and is not easy, and thus is very interesting at the same time.

The aim of the study is to determine the conditions for forming by electrospinning, followed by the formation and systematic characterization of the structure and properties of nonwovens containing poly(glycerol sebacate) and polylactide.

## 2. Materials and Methods

The investigated materials consisted of one component (PLA only) or two components (PLA and PGS). In the case of one component material, the name is straight, for example, PLA_24h, where “24h” means that sample was treated in crosslinking conditions for 24 h. If there are two components in the material, the sample name may look like PLA5050LprePGS or PLA5050HprePGS_3h, which means these samples consist of 50% of PLA and 50% of PGS (Lpre or Hpre type, explained in Section 2.1) and the latter was treated in crosslinking conditions for 3 h. PLA was bought from Corbion (PL49 resomer). Its molecular weight was measured, both M_n_ and M_w_, using the gel permeation chromatography (GPC) method, and the values were 186,000 Da and 490,000 Da, respectively.

### 2.1. Synthesis of Two Types of PGS 

**Figure 1:**
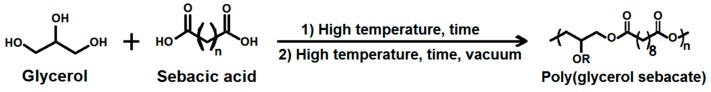
Scheme for two-step poly(glycerol sebacate) (PGS) synthesis.

PGS was obtained by polycondensation of sebacic acid and glycerol—two monomers approved by the U.S. Food and Drug Administration (FDA). Synthesis of PGS consists of two major steps—prepolymerization and crosslinking (Figure 1).

The temperature and time of both steps, as well as the pressure of the crosslinking step, can be adjusted, leading to various properties [2,3].

Prepolymer is a result of the prepolymerization stage, when the linear chains between monomers are formed as a result of esterification between glycerol and sebacic acid. Initiators of the reaction are the hydroxyl groups of the polyol. The primary hydroxyl group of glycerol grasps a carboxyl group to form a monoester with a free carboxyl group and water as a byproduct. Subsequently, this monoester reacts with another primary group of glycerol to form another monoester, and it continues with all available functional groups. Next, curing is required to evaporate glycerol and water residues, enabling PGS to form a stiffer, crosslinked network.

Two types of poly(glycerol sebacate), differing with esterification degree (lower, termed LprePGS, and higher, termed HprePGS) were synthesized from anhydrous glycerol (Sigma Aldrich, Schnelldorf, Germany, 99%, reagent for molecular biology) and sebacic acid (Sigma Aldrich, 95%). Also, in both cases, synthesis was carried out in MultiMax reactors system. Glass reactors of 50 mL with starter, reflux condenser, and temperature sensor were used. Because of the use of such advanced equipment, our experiments are highly repeatable. These syntheses are also solvent- and catalyst-free.

**LprePGS synthesis**

Sebacic acid (3000 g; 0.15 mol) and anhydrous glycerin (27, 26 g; 21, 65 mL; 0.,30 mol) were weighed into a 50 mL glass reactor. Polycondensation was carried out at 150 °C for 24 h. After the reaction, the product was cooled to room temperature and dissolved in acetone. Then, pure prepolymer was obtained by simple precipitation in water and filtration. After that, prepolymer was dried under the fume hood for about 24 h. Pure LprePGS is a white, solid wax.

**HprePGS synthesis**

To obtain prePGS with a higher esterification degree, two-step polycondensation has to be done. The first step is similar to LprePGS synthesis. In a 50 mL glass reactor, sebacic acid (2500 g; 0.12 mol) and anhydrous glycerin (1137 g; 9.03 mL; 0.12 mol) were weighed. The first step of polycondensation was carried out at 150 °C for 24 h. Next, the temperature was increased to 170 °C for 10 min. After that, the second step was started in the same flask, without adding any monomers or catalysts. The second step was carried out at 170 °C for 4 h under 200 mbar pressure. The purification method is similar to the method for LprePGS (see above), but in this case, longer drying, for about 72 h, is recommended. During drying, it is possible that acetone would sweat from polymer, which is a normal phenomenon, which depends on the speed of prepolymer precipitation. It does not affect the final product purity. Pure HprePGS is a yellowish, sticky elastomer.

#### 2.1.1. Nuclear Magnetic Resonance (NMR) Spectroscopy

Agilent 400 MHz spectrometer was used to obtain the 1H, correlation spectroscopy (COSY), and total correlation spectroscopy (TOCSY) spectrum. Deuterated dimethyl sulfoxide (DMSO) was used as a solvent. NMR spectroscopy was used for the determination of polymer parameters such as the polymerization degree, degree of branching, and for polymer chain structure analysis. NMR spectroscopy allows to observe any signals from monomers, so potential impurity due to the presence of monomers in the final product could be estimated.

#### 2.1.2. Esterification Degree

Esterification degree (ED) was calculated on the basis of the following formula:

(1) $$ED=\frac{EN}{EN+AN}\times 100\%,$$

where *ED*—esterification degree, *EN*—ester number, and *AN*—acid number.

Acid number (*AN*) was determined by the simple titration method. The sample was titrated 0.1 mol sodium base in the presence of thymol blue. Acid number was calculated by the following formula:

(2) $$AN\left[\frac{mgKOH}{g}\right]=\frac{\left(v-v_{0}\right)\times M_{NaOH}\times 56.1}{m},$$

where *AN*—acid number (mgKOH/g sample); *v*_0_—volume of NaOH used to titrate the blank test; *v* —volume of NaOH used to titrate the sample; *M_NaOH_*—titre of *NaOH*; KOH has a molar mass of 56.1; and m—mass of the sample.

**Ester number (EN)**, as previously described, was examined by the titration method. Firstly, the sample was boiled with a known amount of 0.1 M potassium base under reflux for 1 h. After that, the excess of added KOH 0.1 M was titrated with hydrochloric acid in the presence of phenolphthalein. Ester number was calculated by the following formula:

(3) $$EN\left[\frac{mgKOH}{g}\right]=\frac{\left(v_{0}-v\right)\times M_{HCl}\times 56.1}{m}-AN,$$

where *AN*—acid number (mgKOH/g sample); *v*_0_—volume of *HCl* used to titrate the blank test; *v*—volume of *HCl* used to titrate the sample; *M_HCl_*—titre of *HCl*; KOH has a molar mass of 56.1; and *m*—mass of the sample.

#### 2.1.3. Molecular Weight

Assuming a linear structure of both PGS prepolymers, the esterification degree can be treated as monomers degree of conversion. The number average molecular weight can be determined from the following equation:

(4) $${\bar{M}}_{n}=M_{0}\times \frac{1}{1-ED},$$

where ${\bar{M}}_{n}$—average molecular weight, *M*_0_—molecular weight for repetitive structure, and *ED*—esterification degree.

Also, the weight average molecular weight can be determined using the following formula:

(5) $${\bar{M}}_{w}=M_{0}\times \frac{1+ED}{1-ED},$$

where ${\bar{M}}_{w}$—weight average molecular weight, *M*_0_—molecular weight for repetitive structure, and *ED*—esterification degree.

The polydispersity index (PDI) could be classically determined by the quotient of weight average molecular weight and number average molecular weight.

### 2.2. Electrospinning of PLA–PGS Blends

PGS was electrospun as a blend with PLA using hexafluoroisopropanole (HFIP) as a solvent at three compositions by weights: 75%/25%, 50%/50%, 25%/75%, and 0%/100%. As the viscosity varies for various compositions of polymers blends, concentrations (w/w) of solutions were adjusted in order to obtain similar fibers’ diameters within the same compositions. For blends of PLA with LprePGS, the concentrations used were 4%, 5.8%, and 11%. For blends of PLA with HprePGS, the concentrations used were 3.5%, 4.5%, and 6.5%. The concentration of pure PLA solution was 3%. Solutions were stirred using a magnetic stirrer for 24 h in ambient conditions. The electrospinning process was held using a self-made electrospinning setup in horizontal mode. Electrospinning parameters were set as follows: feed rate 1.2 mL/h, voltage 11 kV, and distance between needle and collector 14 cm. A rotating drum (8 cm diameter) was used as a collector, with a rotation speed of 250 rpm.

### 2.3. Crosslinking of Nonwovens

Crosslinking of nonwovens was performed in a vacuum oven, at 135 °C, 10 mBar vacuum, over various times (3 h, 6 h, 8 h, 18 h, 24 h, 48 h). To check the effectiveness of crosslinking of PGS, three samples of each nonwoven were subjected to leaching in 99.8% ethanol, with stirring, 1 h long at 25 °C. Non-crosslinked PGS, easily soluble in ethanol, was leached out. The percentage of crosslinked PGS, which remained within fibers, was calculated based on mass measurements of the dried samples.

### 2.4. Scanning Electron Microscopy (SEM)

Jeol JSM 6010 microscope was used for nonwovens morphology observations. Nonwovens were sputtered with a thin layer of gold (below 10 nm) and observed using secondary electron imaging mode. Fibers’ diameters measurements were done using ImageJ software. The average values were calculated out of 100 measurements. The same measurements were used to prepare distribution graphs in Origin software.

### 2.5. Wettability

OCA Physics equipment was used, deionized water droplet was put on the surface of materials, and the contact angle was measured after 0.2 s and 10 s. Two times were chosen, as the experience of authors suggested not to classify materials as hydrophobic too soon, even if the contact angle reading after a typical, short time of 0.2 s is above 90 degrees. Especially when it comes to materials with a porous or irregular surface, it very often takes a few seconds to let the droplet start damping the material. Thus, measurement after 10 s is considered by the authors as more reliable, and better defining materials, but measurements after 0.2 s are shown as well.

### 2.6. Differential Scanning Calorimetry (DSC) Analysis

Perkin Elmer Pyris 1 equipment was used, with cooling and heating rate of 10 °C/min. Samples of approximately 4 mg in weight were used. DSC measurements were done to observe the thermal effects both in PLA and PGS in electrospun bicomponent materials, as well as the differences in thermal effects between LprePGS and HprePGS. Heat of fusion for 100% crystalline PLA was taken as 93.6 J/g [32].

### 2.7. FTIR Analysis

Bruker Vertex 70 Attenuated Total Reflectance (ATR) spectrometer was used to obtain IR spectrums for the investigated samples, with a resolution of 1 cm^−1^, and an average of at least eight scans. This method was chosen as a good indicator of differences between LprePGS and HprePGS, as well as changes in its chemical structure (hydroxyl groups significance) owing to the crosslinking process.

## 3. Results and Discussion

### 3.1. PGS Characterization

Both synthesis procedures used to obtain LprePGS and HprePGS were successful, which was confirmed by spectral analyzing methods. Also, both materials were pure, which means that there were no traces of monomers or residual solvents in the materials.

**Figure 2:**
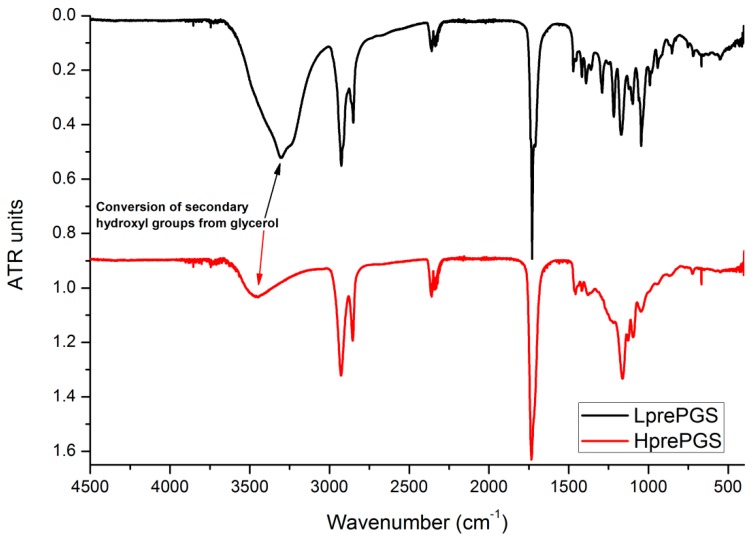
FTIR spectrum for LprePGS and HprePGS. PGS, poly(glycerol sebacate).

FTIR spectrums for both types of PGS indicate small differences, resulting from the various structures of prepolymers. The wide band at 3600–3100 cm^−1^ is much smaller for the H type, which could be related to conversion of secondary hydroxyl groups from glycerol. Also, creation of new ester bonds is confirmed by shifting the peak located around 1730 cm^−1^ from lower values (characteristic for saturated acids) towards higher values (characteristic for saturated esters), but this change is quite tricky to be taken for granted, as that peak is just one of a few appearing in a range of 1730–1750 cm^−1^ (Figure 2). Sharp bands at 3000–2800 cm^−1^ come from C–H bonds. As the chains’ mobility is considered to be lower for the H type PGS, vibration of C–H bonds is also weaker.

In both cases, there are not any signals from monomers at the spectrums that are usually located at 1647 cm^−1^ for glycerol, the maximum of wide band 3200–3500 cm^−1^ groups shifted to 3500 cm^−1^ and 1705 cm^−1^, both latter characteristics for sebacic acid.

**Figure 3:**
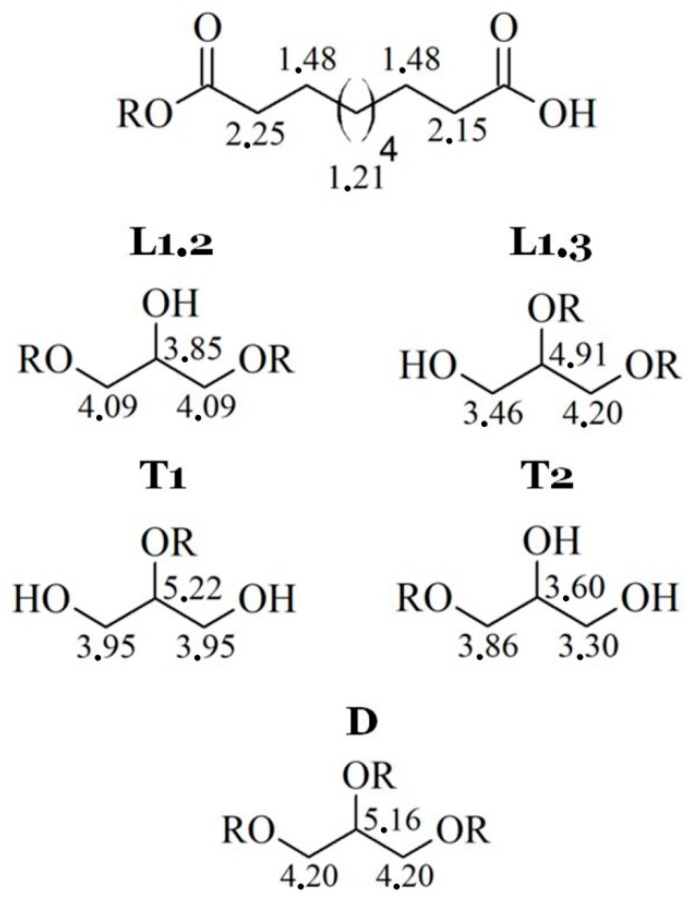
^1^H signals for each spin system in poly(glycerol sebacate).

For both types, the NMR spectra are very similar. Unfortunately, the resolutions of spectra are quite low, but ^1^H signals for each spin system were determined (Figure 3).

**Figure 4:**
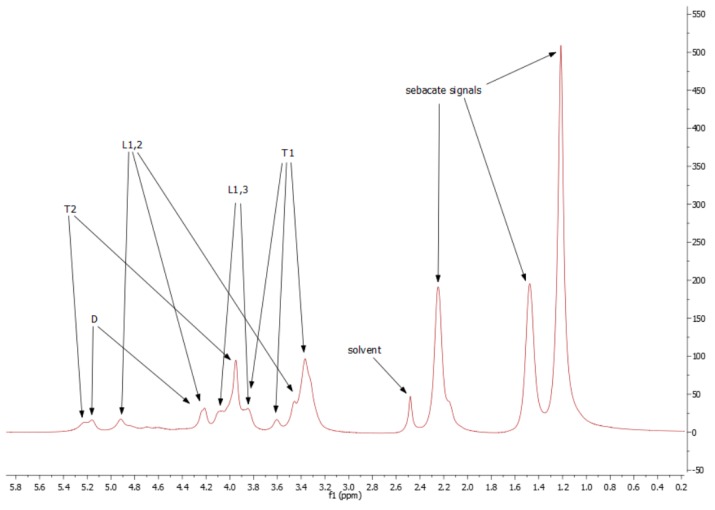
^1^H spectrum for H-prePGS (L-prePGS is very similar).

Because of low resolution and overlapping of the same signals, it is not possible to designate molar mass, PDI, degree of dendrimeryzation, and other parameters. The solution of this problem would be repetition of the NMR experiment on 600 MHz spectrometer, as an example. This is not necessary for aims of this work, because the authors are aware of inaccuracies of designating above parameters from NMR spectra. Moreover, LprePGS and HprePGS are very similar, and the difference between them could not be easily visible by NMR techniques. However, recognition of each signal is very important, because concluding on the basis of the ^1^H spectrum, both prepolymers are pure. In the spectra, there are not any signals of monomers. The ^1^H spectrum is presented in Figure 4.

AN, EN, and ED were determined for both prepolymers. For LprePGS, the values of individual parameters were as follows: AN = 41.2 [mgKOH/g]; EN = 265.4 [mgKOH/g]; and ED = 86%, and for HprePGS, they were as follows: AN = 55.4 [mgKOH/g]; EN = 381.7 [mgKOH/g]; and ED = 87%. The approximate number average molecular weight (Mn) calculated from the above equations is 1971 Da for LprePGS and 2123 Da for HprePGS, and the weight average molecular weight (Mw) is 3666 Da for LprePGS and 3970 Da for HprePGS. The PDIs for both prepolymers are similar (1.86 for L type, 1.87 for H type). The increase of molecular weight and PDI for HprePGS is the effect of the second polycondensation step.

Prepolymer has to be soluble, which is necessary for electrospinning. LprePGS has very good solubility in HFIP, dioxane, acetone, and alcohols. HprePGS also has good solubility in these solvents, but dissolution is much slower than for LprePGS. If the reaction time of the second step for HprePGS would be 1 h longer, then the gelation point would be reached and the polymer would be insoluble.

### 3.2. SEM Nonwovens Morphology Analysis

**Figure 5:**
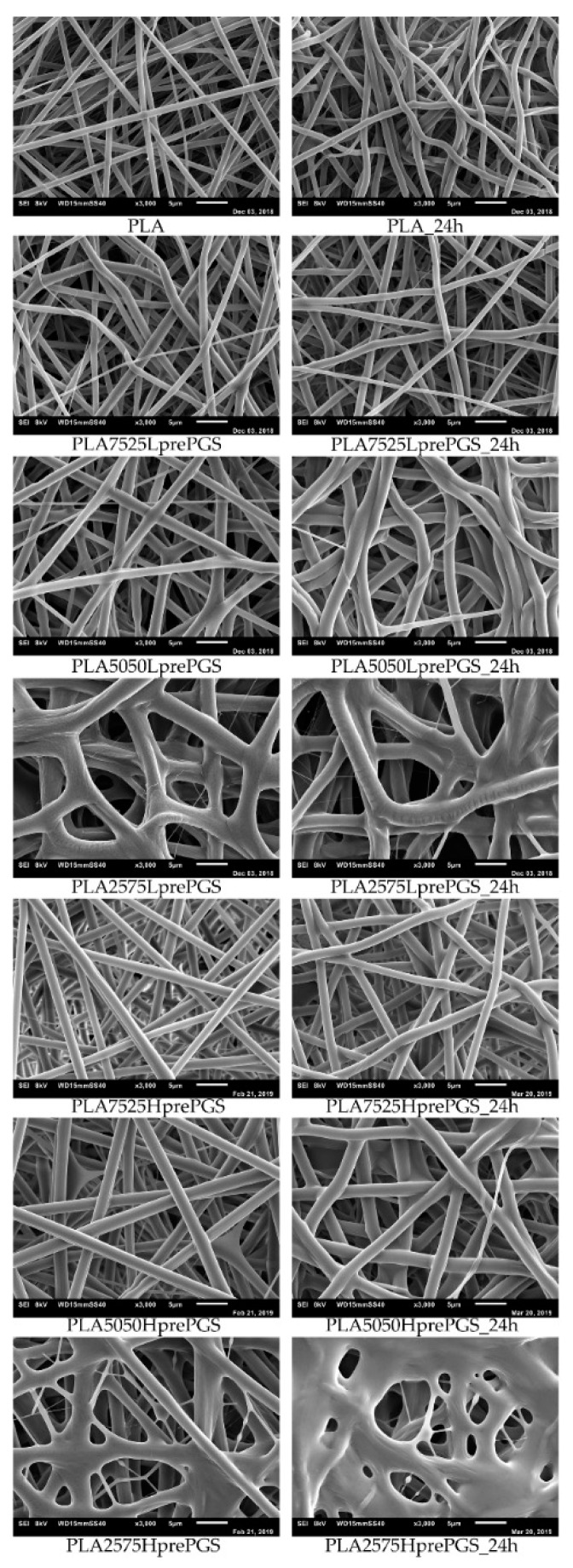
SEM images of electrospun nonwovens, in left column straight after electrospinning, in the right column after 24 h of crosslinking (heating at 135 °C, 10 mBar vacuum).

**Figure 6:**
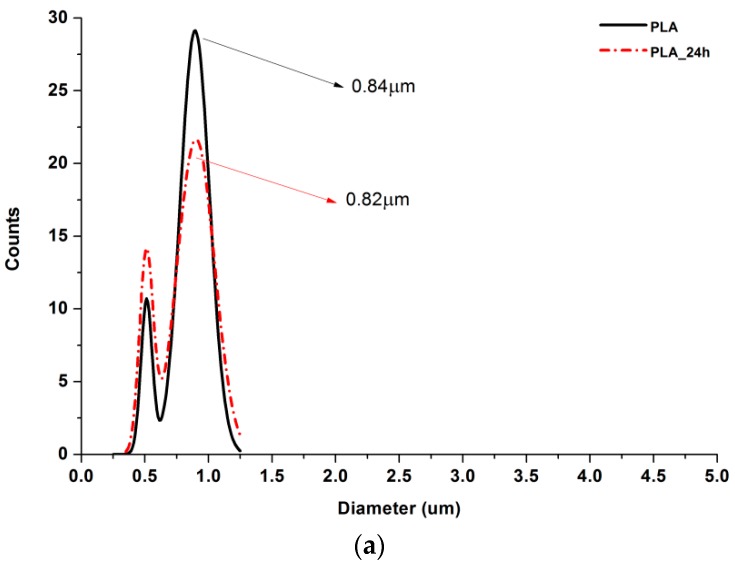

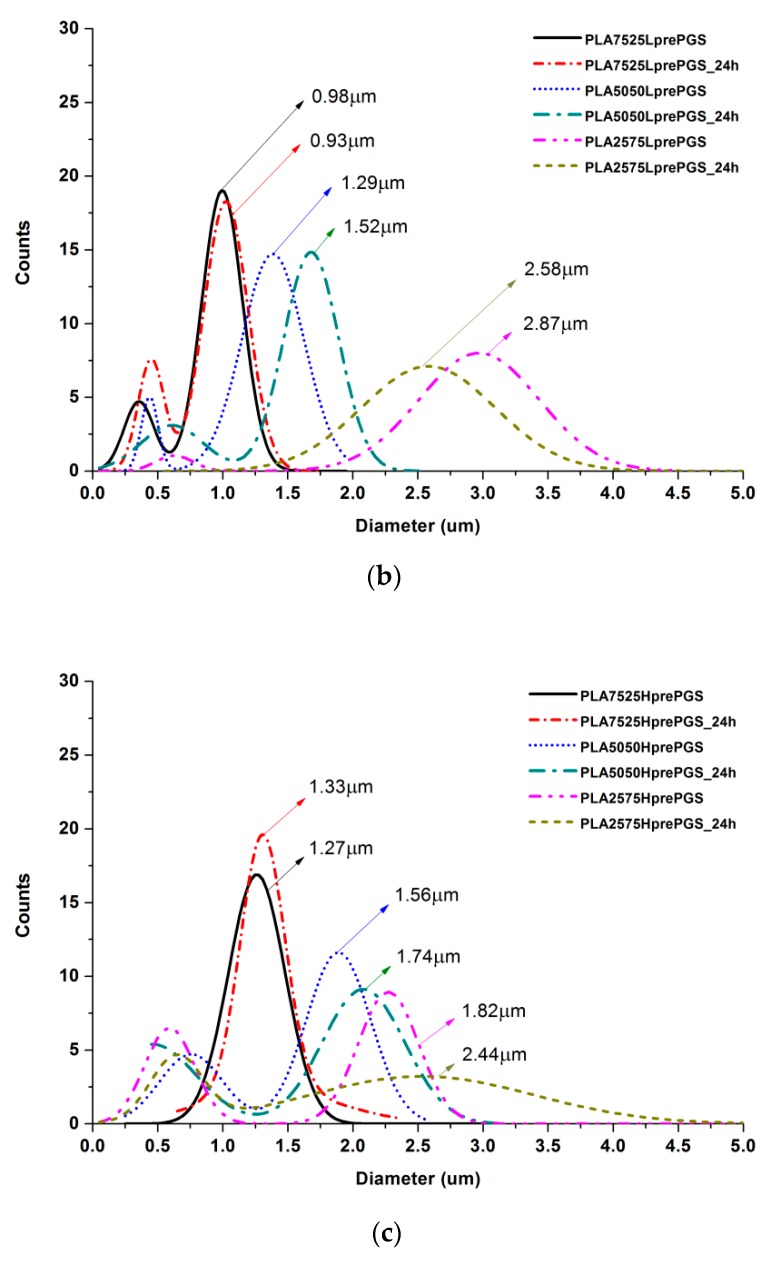
Gauss approximation of fibers’ diameters distributions for investigated samples. (**a**) For samples consisting of PLA only; (**b**) for samples consisting of PLA and LprePGS; (**c**) for samples consisting of PLA and HprePGS. Arrows indicate average values. Measurements for samples with highest PGS content (75%) neglect areas overflowed by PGS and should be considered as a very approximate representation of the whole nonwoven.

According to Figure 5, 25% PGS contribution does not bring much difference in nonwoven morphology, compared with pure PLA fibers. The effect of 50% PGS contribution is clearly visible, especially after the crosslinking process, and the 75% PGS contribution affects the morphology of nonwoven dramatically even without crosslinking—there are many more areas that resemble film, where PGS overflowed PLA fibers. Fibers’ diameters distributions are bimodal (Figure 6), which may come from the strong interaction between the polymer stream jet and electrostatic field, resulting in high instabilities and splitting of the jet into thinner fibers. In the case of compositions with 25% and 50% LprePGS content, the average diameters moved slightly higher. We would associate this fact with the liquid nature of PGS prepolymer in such a high temperature (before crosslinking) and with shrinking of the PLA fibers as a result of the structural relaxation mechanism. The same amount of PGS remains at and within the shrinked fibers of PLA, and thus the resultant diameters are slightly higher. In case of 75% LprePGS content, where the average diameter moved to lower value, it can be associated with something different. For these samples (with the highest content of PGS, Lpre, and Hpre), areas overflowed by PGS were neglected. In the samples treated with a high temperature, before PGS crosslinked thin PLA fibers were revealed, to which PGS adhered after electrospinning. Some of those thin PLA fibers were measured, with a decreasing average diameter.

Typical SEM images of nonwovens with various compositions are shown in Figure 5. 

PLA is not susceptible to the crosslinking process, but the temperature of 135 °C affects nonwovens and polymer in other ways. What is visible without a microscope, is that nonwovens shrink immediately after putting it into temperature higher than the PLA glass transition point, T_g_, which is between 55 °C and 65 °C. Such shrinking affects the morphology of fibers, which is evident from SEM images (Figure 5)—fibers become less straight and more “wavy” after treatment in high temperature, the morphology seems to be “cramped”, and fibers are closer to each other. It is appropriate for all nonwovens, as all of them contain PLA. During the electrospinning process, the structure of fibers is formed very fast, and thus it is very far from thermodynamic equilibrium, resulting in subsequent relaxation/shrinking. Structural relaxation effects in PLA fibers obtained using fast processing methods are still under investigations and are related to thermo-mechanical stability of this polyester [33,34]. Going back to SEM images, the high temperature and vacuum treatment affect the morphology of nonwovens just slightly, yet it is visible, especially for samples that contain 50% and 75% of PGS. Only samples heated for 24 h are shown, because there are no differences in nonwovens’ morphology, regardless of crosslinking time (considering times not shorter than 3 h, shorter times were not investigated), and in the beginning of those investigations, 24 h was considered by the authors as a good reference point for crosslinking time.

### 3.3. Crosslinking Efficiency

**Figure 7:**
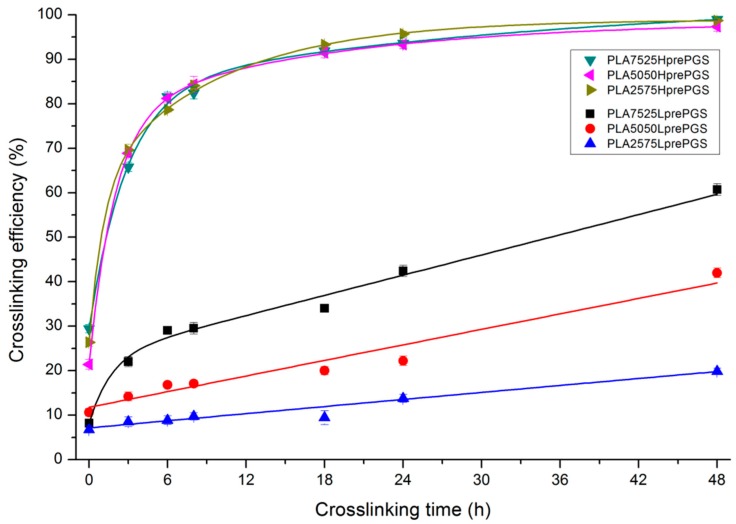
Sol-gel analysis vs. crosslinking time for bicomponent, PL49, and LprePGS or HprePGS nonwovens. The absolute values of the error are plotted and range from 2% to 4%.

The effectiveness of crosslinking of two types of synthesized PGS (LprePGS and HprePGS) in nonwovens can be evaluated from Figure 7, which presents the gel fraction versus crosslinking time.

Susceptibility to crosslinking is much higher for HprePGS compared with LprePGS, and its crosslinking efficiency is independent of the PGS content in nonwoven. LprePGS is much less susceptible to crosslinking and, at the same time, the content of gel is very dependent on PGS content. HprePGS, after 6 h of the crosslinking process, hits 80% crosslinking efficiency, while for LprePGS, after the same time, the crosslinking efficiency values are between 9% and 29%. An 80% level is unattainable even after 48 h, even for the PLA7525LprePGS sample. The more LprePGS within the nonwoven, the more difficult it is to obtain a higher content of crosslinked PGS.

### 3.4. Wettability

**Figure 8:**
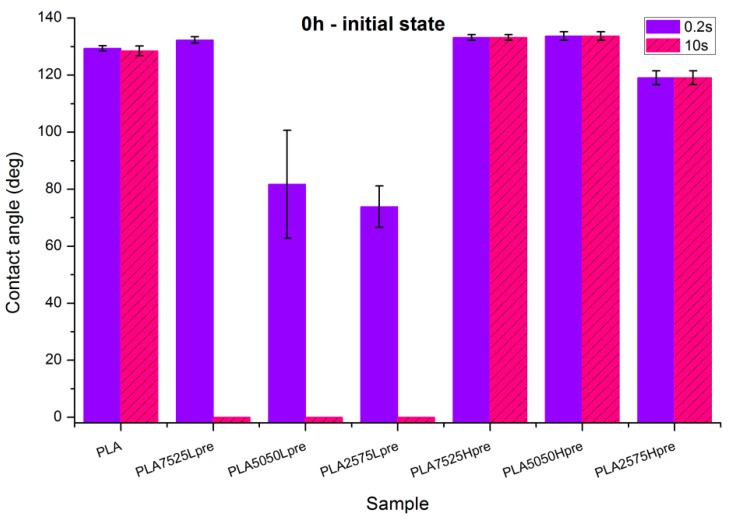
Contact angle measurements for electrospun nonwovens without any additional treatment.

Observations of wettability for electrospun nonwovens before crosslinking (Figure 8).

- PLA is a polyester with contact angle values around 70°–80° for film samples, and contact angle values increase in nonwoven form, which is a known phenomenon [35]
- Nonwovens consisting of PLA and LprePGS are hydrophilic owing to LprePGS, which is a hydrophilic polymer thanks to the –OH groups. The more LprePGS, the more apparent the hydrophilic nature. For all samples with LprePGS, after 10 s, a whole water droplet is adsorbed by the materials
- HprePGS, which is supposed to have much less –OH groups owing to the higher degree of conversion of sebacic acid and glycerol, higher crosslinking degree, and higher esterification degree after the second step of polycondensation, compared with LprePGS, does not bring hydrophilicity to nonwovens. A slightly lower contact angle for the PLA2575Hpre sample (but still in the hydrophobic range) may be because of the higher content of HprePGS, as well as (or even more likely) because of the different morphology of nonwoven compared with samples with a lower HprePGS content.

**Figure 9:**
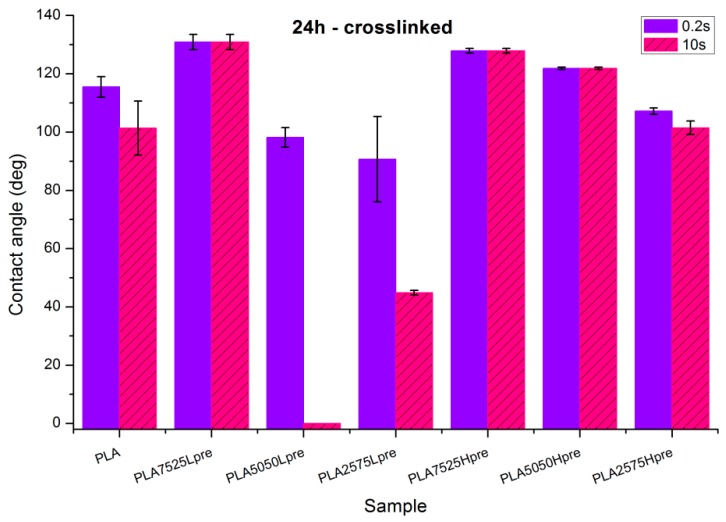
Contact angle measurements for electrospun nonwovens subjected to 24 h long crosslinking in 135 °C and 10 mBar vacuum.

Observations of wettability for electrospun nonwovens subjected to 24 h long crosslinking (Figure 9).

- Nonwoven of pure PLA became slightly less hydrophobic, for 10 s, being not much above the hydrophilic boundary value of 90°. After the crosslinking process, shrinking appears; fibers are closer to each other; and the morphology of the nonwoven is closer to casted film, which is characterized by lower values of contact angle. The difference between the contact angle values for 0.2 s and 10 s also indicates that the water droplet is being adsorbed by the material, slowly but constantly. Such behavior indicates that the material is rather hydrophilic than hydrophobic, even if the contact angle values at the beginning are above 90 degrees.
- Nonwovens with LprePGS, after 24 h of crosslinking, changed as expected. The blend with 25% addition of LprePGS, with the highest crosslinking degree, became hydrophobic, and the hydrophilicity of other two blends with a higher content of LprePGS was altered toward higher contact angle values, owing to the diminishing of free –OH groups’ content.
- For nonwovens with HprePGS, the contact angle values slightly decreased, but remained in the hydrophobic range. Observations may indicate that, when it comes to the hydrophobic materials analyzed here, the change in the morphology of nonwoven after heating (shrinking) may lead it to less hydrophobic properties, just as was observed for pure PLA nonwoven.

**Figure 10:**
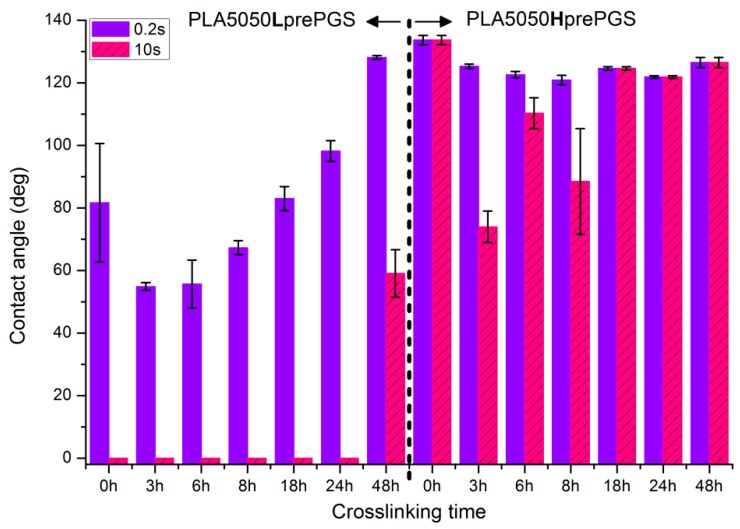
Contact angle measurements for electrospun nonwovens consisting of 50% PLA and 50% PGS (Lpre or Hpre) subjected to various times of crosslinking (from 3 h to 48 h) in 135 °C and 10 mBar vacuum. In the figure, 0 h corresponds to samples not subjected to crosslinking.

Figure 10 illustrates the systematic analysis of wettability changes with crosslinking time for electrospun nonwovens consisting of 50% PLA and 50% Lpre or Hpre PGS.

- For nonwovens with LprePGS, with the shortest time of crosslinking, during which LprePGS is crosslinked just slightly, the major effect of increasing hydrophilicity probably comes from the change in morphology of nonwoven—shrinking and reduced spaces between fibers. Longer times of crosslinking lead to increasing of the contact angle registered at 0.2 s. Nonwovens, however, remain hydrophilic even after 48 h of crosslinking
- For nonwovens with HprePGS, similar effects are observed, but within the range of much higher values of the contact angle; after 3 h of crosslinking, a decrease in the contact angle is observed compared with non-crosslinked nonwoven—because of the change of nonwoven morphology, which overcomes influence of increasing the crosslinking degree. After longer times of crosslinking, however, the contact angle values increase, especially those registered after 10 s—nonwovens become more and more hydrophobic up to 18 h of crosslinking. After that, with the crosslinking degree of SC PGS at the level of 90% and above, the contact angle stays above 120 degrees, more or less within the error range.

### 3.5. DSC Analysis

**Figure 11:**
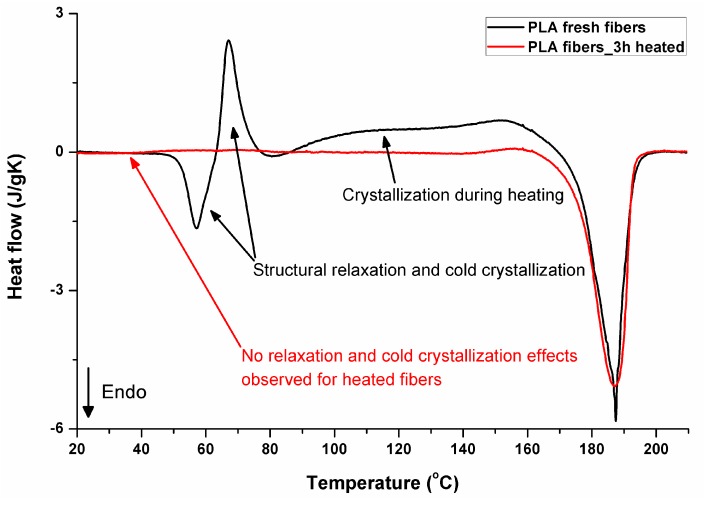
DSC first heating, fresh and “relaxed” (heated for 3 h at 135 °C after electrospinning) PLA fibers.

Figure 11 illustrates the thermal effects during the first heating of PLA. The thermograms of electrospun fibers start with structural relaxation at 57 °C (endothermic peak), followed immediately by cold crystallization seen as the exothermic peak at 67 °C, and subsequent crystallization during further heating in the range from 86 °C to around 169 °C. The melting occurs in the temperature range from 169 °C to 195 °C. It should be noted that the melting (52.7 J/g) includes crystals formed during the electrospinning process (during evaporation of solvent), cold crystallization (13.9 J/g), and the broad crystallization effect between cold crystallization and melting (35,8 J/g). From the balance of the area of the exothermic crystallization peaks during heating and the area related to the melting, it is evident that the original crystallinity was only 3.2% (3 J/g). Extremely fast evaporation of solvent during electrospinning is a key factor responsible for crystallization hindering. As the process takes place in ambient conditions, below a glass temperature of PLA, relaxation mechanisms are rapidly activated after heating material in the glass transition region. The endothermic effect in glass transition temperature region is related in the literature, for example, the works of [36,37,38,39,40], to structural enthalpy relaxation, after which a cold crystallization exothermic peak may appear. It does not exhaust the structure completely—it is still able to significantly crystallize during further heating before melting. That effects are not observed during DSC heating in materials that were heated before DSC analysis, allowing earlier structure relaxation. The 3 h heated sample is presented, as it is the shortest time investigated by the authors, and it is already enough to “relax” the structure of the material. According to other investigations by the authors [33], even shorter times, of just a few minutes of heating above the PLA glass transition region, would be enough to “relax” the structure of the material analyzed. For such relaxed samples, crystallinity determined from the area of melting peak was 66.5% for the sample heated for 3 h at 135 °C, reaching 72% after keeping it for 24 h at 135 °C. The thermal effects in PLA that was processed using rapid methods with very fast structure formation during very fast solvent evaporation (solution electrospinning) or cooling from a melt below glass transition have been under investigations for years, and just two, among many other publications about that phenomenon, have already been cited [33,34].

**Figure 12:**
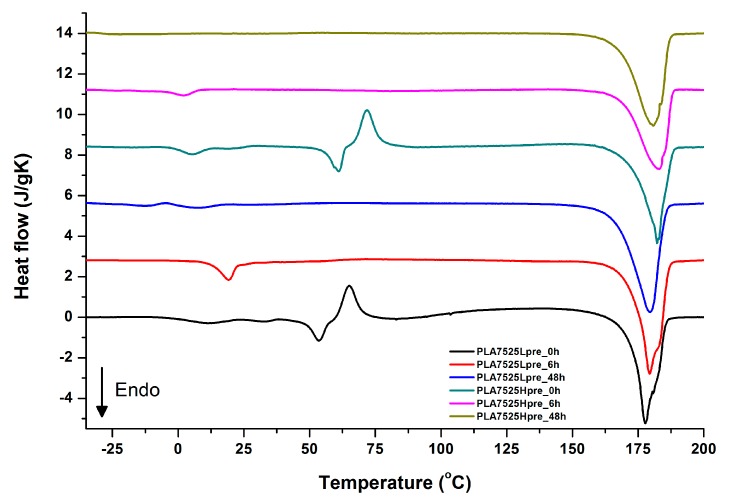
DSC first heating of nonwovens with 75% PLA and 25% PGS (Lpre or Hpre), crosslinked for 0 h (initial state), 6 h, and 48 h.

**Figure 13:**
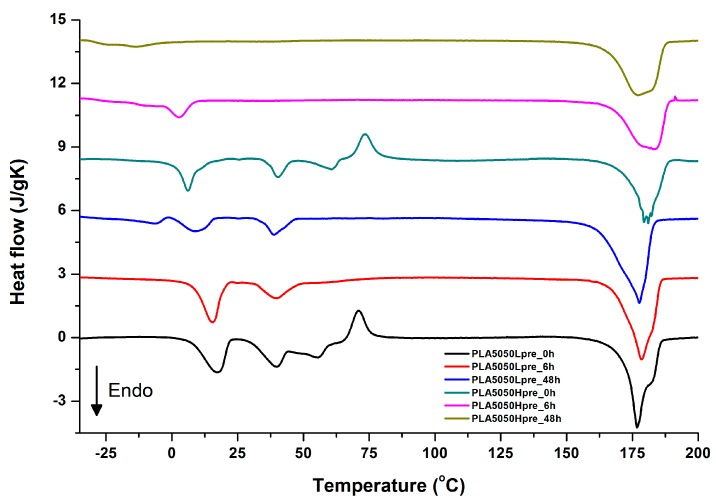
DSC first heating of nonwovens with 50% PLA and 50% PGS (Lpre or Hpre), crosslinked for 0 h (initial state), 6 h, and 48 h.

**Figure 14:**
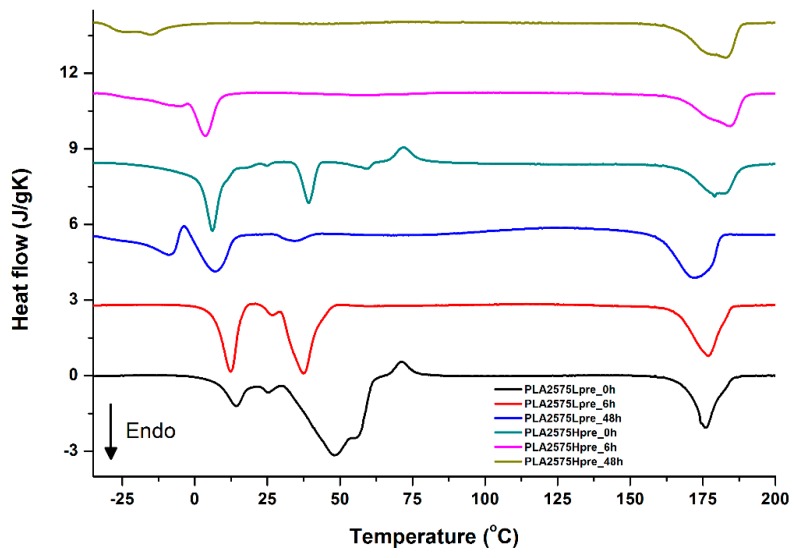
DSC first heating of nonwovens with 25% PLA and 75% PGS (Lpre or Hpre), crosslinked for 0 h (initial state), 6 h, and 48 h.

**Figure 15:**
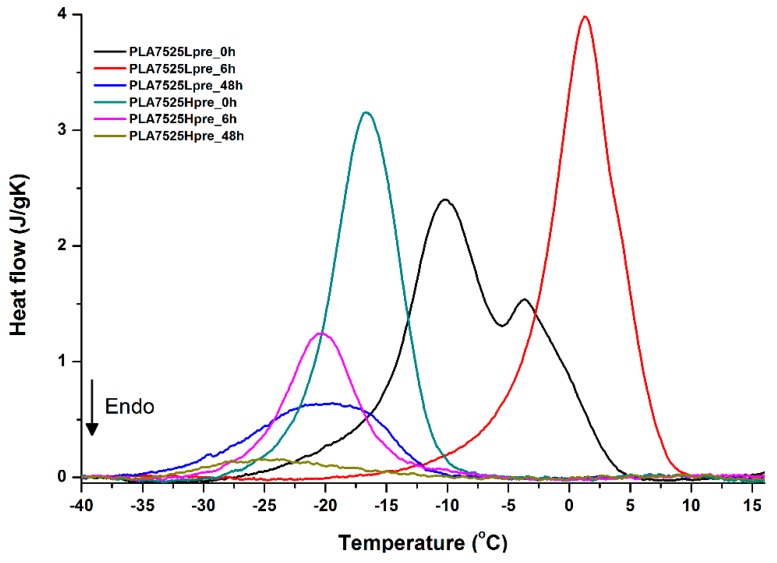
DSC first cooling of nonwovens with 75% PLA and 25% PGS (Lpre or Hpre), crosslinked for 0 h (initial state), 6 h, and 48 h.

**Figure 16:**
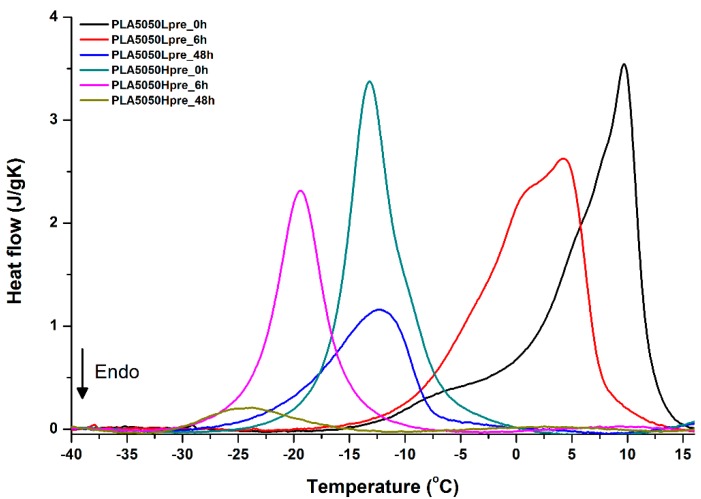
DSC first cooling of nonwovens with 50% PLA and 50% PGS (Lpre or Hpre), crosslinked for 0 h (initial state), 6 h, and 48 h.

**Figure 17:**
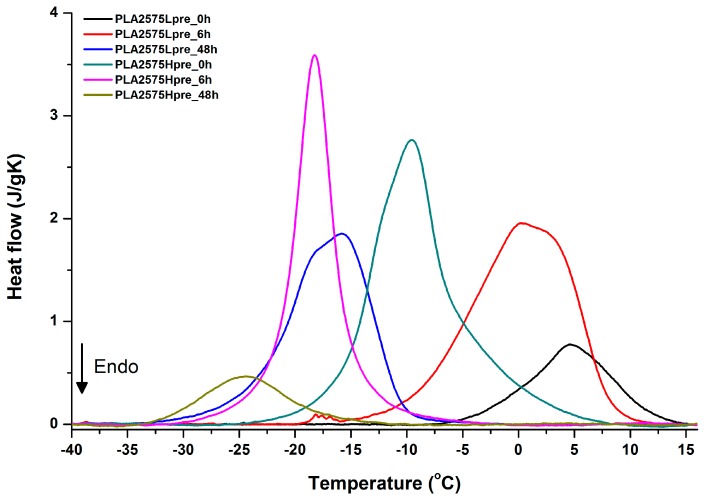
DSC first cooling of nonwovens with 25% PLA and 75% PGS (Lpre or Hpre), crosslinked for 0 h (initial state), 6 h, and 48 h.

For selected bicomponent nonwovens, first heating (Figure 12, Figure 13 and Figure 14) followed by cooling (Figure 15, Figure 16 and Figure 17) scans are presented. First heating scans provide information about the phase transition effects of PGS and PLA, as well as about possible structure relaxation in PLA. First cooling characterizes the effect of crystallization ability of PGS in those particular conditions.

It is evident from Figure 12, Figure 13 and Figure 14 that the heating of fibers starts with structure changes in PGS in the temperature range between −25 °C and 25 °C for a lower PGS content, and extending to even 40 °C for a higher PGS content. Those heat effects are relatively complex, starting most probably with glass transition followed by the endothermic effect of crystal melting. This interpretation is in accordance with previous results [3,41] indicating T_g_ in a range of −35 °C to 15 °C and T_m_ in a range −12 °C to 2 °C for PGS, which depend on the prepolymer esterification degree and crosslinking degree, and may vary even more. Considering such an interpretation of low temperature DSC effects, it is seen from Figure 12, Figure 13 and Figure 14 that they are more pronounced for fibers with a higher PGS content, even after normalization to the mass of the PGS component in the sample. Moreover, in the case of the higher PGS content (Figure 13 and Figure 14), there is an additional endothermic effect of crystal melting at ca. 40 °C. It is anticipated by us that there is some miscibility of PGS and PLA, leading to the formation of various mixed crystals with different melting temperatures. The effect of crosslinking on PGS can be deduced from reduction of the heat, as well as the temperature of melting with the increasing time of crosslinking. This indicates lower crystallinity and smaller/more defective crystals for the less mobile crosslinked structure of PGS. In the case of heat effects related to PLA, there is in general glass transition, followed by cold crystallization and crystal melting, as was observed for pure PLA. For PLA that does not undergo crosslinking, an increase of the crosslinking time results only in structure relaxation manifested by the lack of heat effects immediately after the PLA glass transition point. It is seen from Figure 13 and Figure 14 that 6 h at 135 °C is absolutely enough to eliminate the relaxation effects in PLA. Moreover, an increase of crosslinking time leads to growth/perfecting of PLA crystals manifested by a slight increase of crystal melting temperatures. The last observation drawn from DSC heating thermograms of fibers is that the shape of the PLA melting endotherm is bimodal, being a support of some PLA–PGS miscibility anticipation and formation of mixed crystals. 

Crystallization of PGS can be observed in Figure 15, Figure 16 and Figure 17, where first cooling scans are presented. For PLA5050PGS samples, it is clearly visible that, with the increasing time of crosslinking, the crystallization ability decreases, which is manifested by the reduction of crystallization heat as well as crystallization temperature. For PLA7525PGS and PLA2575PGS samples, the general impression is similar. The observed effect of crosslinking time on crystallization ability is related to molecular mobility. Bimodal crystallization peaks correspond well with double melting peaks that occur during heating below 50 °C (Figure 12, Figure 13 and Figure 14), and are most probably related to the miscibility (limited) in the PLA–PGS system, leading to the formation of mixed crystals. The higher the crosslinking degree of PGS, the more homogeneous the structure. For samples with crosslinking efficiency higher than 80% (according to Figure 7, samples with HprePGS crosslinked for 6 h or longer), single peaks are observed for both crystallization and melting effects of PGS.

### 3.6. FTIR

**Figure 18:**
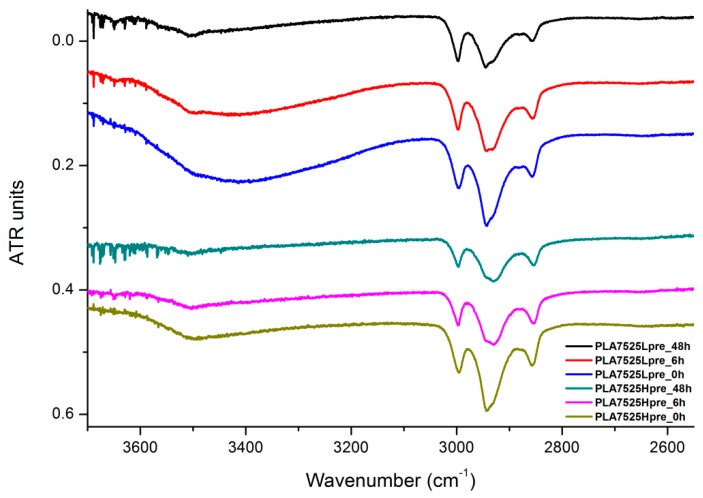
FTIR spectrum of nonwovens with 75% PLA and 25% PGS (Lpre or Hpre), crosslinked for 0 h (initial state), 6 h, and 48 h.

**Figure 19:**
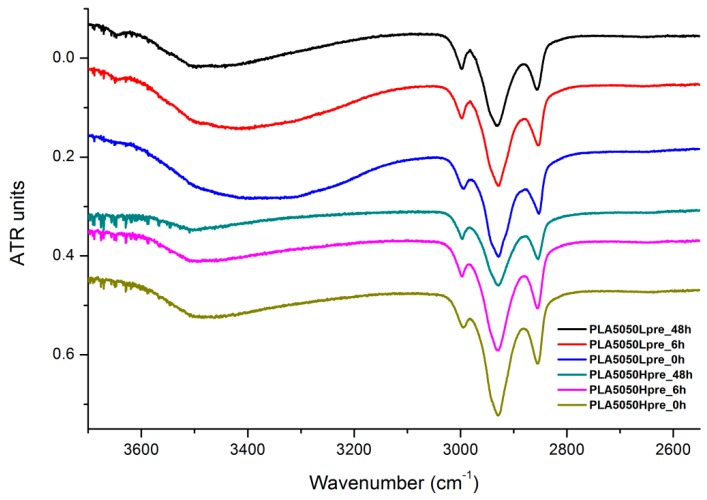
FTIR spectrum of nonwovens with 50% PLA and 50% PGS (Lpre or Hpre), crosslinked for 0 h (initial state), 6 h, and 48 h.

**Figure 20:**
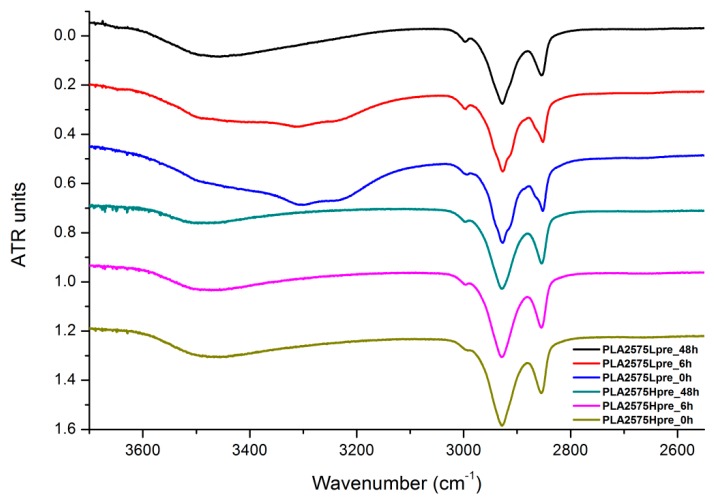
FTIR spectrum of nonwovens with 25% PLA and 75% PGS (Lpre or Hpre), crosslinked for 0 h (initial state), 6 h, and 48 h.

The presented results (Figure 18, Figure 19 and Figure 20) show the effect of increasing the crosslinking time, which affects the wide band at 3600–3100 cm^−1^, which characterizes the –OH groups, and sharper bands at 3000–2800 cm^−1^, which come from the C–H bonds, from both PLA (the band closest to 3000 cm^−1^ and one component of the bimodal middle band) and from PGS (the band closest to 2800 cm^−1^ and one component of the bimodal middle band). The hydroxyl group band diminishes considerably, when comparing 0 h, 6 h, and 48 h samples with increasing time. The smallest band is observed for the 48 h samples, which could be related to the conversion of secondary hydroxyl groups from glycerol—diminishing of the –OH groups, which can be easily related to the contact angle results, where a higher crosslinking degree meant a shift toward hydrophobicity. Several bands between 2800 cm^−1^ and 3000 cm^−^1, which come from C–H bond vibrations, diminish with crosslinking time, owing to the constricted mobility in crosslinked PGS and in PLA crystallized to a higher degree.

## 4. Conclusions

All of the above investigations were done on a way to obtain bicomponent nonwovens with increased elasticity compared with pure PLA nonwovens. The presented work confirms the successful synthesis of two types of pre-PGS; shows the successful electrospinning of bicomponent polymeric system; and demonstrates differences in crosslinking characteristics of two types of pre-PGS within the nonwovens, as well as its influence on the fundamental structural analysis results. This paper does not show mechanical tests, which are planned to be investigated as a next step, but for now, it is already known that PGS, in both variations (Lpre and Hpre), does not provide elasticity to nonwovens. Elasticity was expected after crosslinking of PGS, being a component of nonwovens. Observations at the macroscopic level indicate that the obtained materials do not have the capability for reversible deformations. The thoughts of the authors oscillate around the matter, that the choice of these particular two polymers, with such distinct differences in mechanical characteristics, may work in favor of that polymer that is better in general for processing by the electrospinning method. PLA, a carrier polymer, with thermoplastic properties, much higher molecular weight, and much higher Young modulus and tensile strength, compared with PGS, dominates with its properties, even if its content is just a 25%. Presented, simplified structural studies were done to understand better the structural behavior of that bicomponent system, and decide what steps may be taken to utilize the potential of elastic properties of PGS in bicomponent, polymeric, electrospun systems. From the authors’ perspective, in the end, it would be good to obtain elastomeric nonwovens with all investigated PGS contents. At this stage, it is not clear if any of the used compositions are anywhere closer to that goal. The best crosslinking effectiveness is offered by the compositions with 25% PGS content, but at the same time, such a content may be not enough to diversify mechanical properties of PLA, which is, in theory, mechanically completely different than PGS.

## Figures and Tables

**Table 1:** Potential biomaterials used in cardiac muscle engineering [6]. Reprinted from Biomaterials, 29 (1), Chen, Q.Z.; Bismarck, A.; Hansen, U.; Junaid, S.; Tran, M.Q.; Harding, S.E.; Ali, N.N.; Boccaccini, A.R., Characterization of a soft elastomer poly(glycerol sebacate) designed to match the mechanical properties of myocardial tissue, 47–57, Copyright (2008), with permission from Elsevier. PGA, polyglycolide; PLLA, poly(l-lactide); PGS, poly(glycerol sebacate).

Polymer	Elastomer (E) or Thermoplastic (T)	Young Modulus (Or Stiffness)	Tensile Strength	Degradation (Month)
PGA	T	7–10 GPa	70 MPa	2–12
PLLA or PDLLA	T	1–4 GPa	30–80 MPa	2–12
PHB	E	2–3 GPa	36 MPa	Degradable
PPD (also called PDS)	E	0.6 GPa	12 MPa	6
TMC	E	6 MPa	12 MPa	Degradable
TMC-PDLLA (50:50)	E	16 MPa	10 MPa	Degradable
POC	T	1-16 MPa	6.7 MPa	Degradable
PGS	E	0.04–1.2 MPa	0.2–0.5 MPa	Degradable
Collagen fibre (Tendon/cartilage/ligament/bone)	E	2–46 MPa	1–7 MPa	Degradable
Collagen gel (calf skin)	E	0.002–0.022 MPa	1–9 kPa	Degradable
Myocardium of rat	E	0.001–0.14 MPa	30–70 kPa	NA
Myocardium of human	E	0.02–0.5 MPa	3–15 kPa	NA
